# Developmental Traits of Impulse Control Behavior in School Children under Controlled Attention, Motor Function, and Perception

**DOI:** 10.3390/children8100922

**Published:** 2021-10-16

**Authors:** Hsin-Yung Chen, Ling-Fu Meng, Yawen Yu, Chen-Chi Chen, Li-Yu Hung, Shih-Che Lin, Huang-Ju Chi

**Affiliations:** 1 Department of Occupational Therapy & Graduate Institute of Clinical Behavioral Sciences, College of Medicine, Chang Gung University, Taoyuan City 33302, Taiwan; hychen@mail.cgu.edu.tw; 2 Department of Neurology and Dementia Center, Taoyuan Chang Gung Memorial Hospital, Taoyuan City 33378, Taiwan; vivianchi@adm.cgmh.org.tw; 3 Division of Occupational Therapy, Department of Physical Medicine and Rehabilitation, Chiayi Chang Gung Memorial Hospital, Puzi City 613016, Taiwan; apirlline@cgmh.org.tw; 4 Department of Occupational Therapy, Colorado State University, Fort Collins, CO 80523-1573, USA; yawen.yu@colostate.edu; 5 Health Center, Taipei Fushing Private School, Taipei City 106343, Taiwan; julia04280@gm.fhjh.tp.edu.tw; 6 Department of Special Education, National Taiwan Normal University, Taipei City 106308, Taiwan; t14010@ntnu.edu.tw; 7 College of Teacher Education, National Taiwan Normal University, Taipei City 106308, Taiwan

**Keywords:** impulsive behavior, inhibition, developmental trait, school age

## Abstract

This research surveyed the characteristics of the developmental traits of impulse control behavior in children through parent-report questionnaires. After matching for gender and attention behavior, as well as controlling for variables (motor and perception) which might confound impulse control, 710 participants (355 girls and 355 boys; grade, 1–5; age, 7–12 years) were recruited from a database of 1763 children. Results demonstrated that there was a significant difference between grade 1 and grade 5 in impulse control. Conversely, no significant differences were found when comparing other grades. The present findings indicate that a striking development of impulse control occurs from grade 4 to 5. Moreover, the plateau of impulse control development from grade 1 to 4 implies that a long transition period is needed to prepare children to develop future impulse control. In conclusion, the age-dependent maturation associated with stage-wise development is a critical characteristic of impulse control development in school age children. Further discussions are made regarding this characteristic, such as from the perspective of frontal lobe development.

## 1. Introduction

Impulsivity is considered a characteristic of human behavior that can be beneficial for seizing a valuable opportunity, or for making a disastrous decision, in the rhythm of daily life [1]. In response to the development of impulse control, it is important to develop novel approaches to acquiring and maintaining competence and to rigorously process the influx of information obtained from the environment through appropriate orientation without conflicting behaviors. Although impulsivity can be considered a dimension of personality, children with difficulties in impulse control, particularly those accompanied by attention and perceptual motor impairment, are at risk of a diverse range of behavioral, social, emotional, and academic problems [2,3,4,5,6]. Consequently, each problem indicator interferes with positive adaptation and adjustment in late childhood, adolescent, and adult periods [7,8,9,10].

Measures of age-related differences in brain function are important to understanding the changes in impulse control in developing children. Studies have reported the critical role of brain networks in age-related impulse control by conducting neuropsychological tasks requiring the participants to actively inhibit automatic responses. The Stroop task, for example, involves control processes for participants to actively inhibit their automatic behaviors in conflicting ink color–word reading [11,12]. The neuroimaging results of this task revealed age-related improvements in the inferolateral portion of the prefrontal cortex and lenticular nucleus in developing children [11,13]. In accordance with the neuroimaging results, bodies of neurophysiological evidence have shown that the stop signal task, which may be activated very early (approximately 200 ms) to trigger inhibitory control processes [14], indicated increases in prefrontal activity with both age and impulse control in children [11,14]. In summary, impulse control is considered to improve with age and along with the development of the prefrontal cortex [15,16]. The developmental trait from both neuroimaging and neurophysiological studies indicates that age-related changes in brain network activities underlie the improvements in impulse control and characteristics of normal development trends of humans [11,13,17].

Improvements in the control and regulation of impulsive behaviors are the primary developmental trait of children during the first decade of life [6,18]. These improvements, associated with age or the maturation level, are involved in controlling emotions, positively interacting with others, avoiding inappropriate or aggressive actions, and becoming an autonomous learner [9,18,19]. The developmental trend of these changes first advances from rudimentary types of reflexive regulation in infancy to nascent attempts at volitional behavior control in toddlerhood to reflective self-regulation of behavior in childhood [8,18,20], and then expands to goal-directed self-organized behavior in adolescence [6,21,22]. Precisely, the greatest improvement in impulsive errors and missed responses was observed in three stages of maturation: early childhood (6–8 years), middle childhood (9–12 years), and adolescence (13–15 years) [10,23,24]. Moreover, notably, little or no further improvement was observed in adolescence [24].

Dysfunction in the described characteristic developmental processes of impulse control has been reported to be associated with difficulties in inhibitory control, intolerance to delayed rewards, and slow engaging with decision-making because of the lack of consideration of information alternatives [25,26], as well as more universal deficits such as short attention span, poor perceptual motor abilities, and hyperactivity [3,27,28]. Although the developmental trait of impulse control has been characterized, confounding effects, including attention, perception, and motor function, which influence children’s capacity at each developmental stage, have engendered wide variations in the perception of academic efficacy as well as the use of learning and teaching strategies in school children [2,25,29]. According to our review of the relevant literature, relatively scarce theoretical studies have controlled for the confounders. Developmentally, both fine and gross motor abilities have been suggested as critical elements of development and learning competencies in school-age children. For more than a century, school-age children with motor problems have been associated with behavioral difficulties including inattention, inappropriate overactivity, and problematic impulsiveness [28,30]; moreover, they were reported to have a very high risk of persistent problems in various types of functional performance that translate throughout childhood, adolescence, and adulthood [31].

Previous studies demonstrate that dysfunction in school children’s impulse control is typically assessed through laboratory testing with a certain degree of predictive validity [13,14]. Although laboratory testing provides quantitatively clinical and research recommendations of impulse control, it may encounter difficulties in adequately reflecting most school children’s behavioral tendencies associated with academic and contextual performance in the classroom and daily activities. Parents are typically observed to provide more accurate information about their children’s behavioral problems. Therefore, parent reports provide opportunities, containing substantial objective validity for retrieving direct information as well as contextual demands, for understanding the development characteristics of impulse control in children [18,32]. The present survey study investigated the characteristics of developmental traits of impulse control behavior to determine the importance of behavioral problems and competencies reported by parents. In summary, the two research questions are outlined as follows:

1. How does the trait of impulse control develop in school children?
2. How were the grade/age-related differences in impulse control observed?

## 2. Materials and Methods

### 2.1. Participants

Participants were recruited from a public elementary school in Taoyuan, Taiwan. A total of 1208 questionnaires were completed by caregivers of children in grades 1–5. The questionnaire data were the same as those used by a previous study to conduct a factor analysis of fine motor items [33]. The data from 34 children who were suspected as experiencing delay, as mentioned by class teachers, were not analyzed. Thus, the initial sample included 1174 children in grades 1–5.

Moreover, participants with missing data who could not be accurately classified were excluded from the sample. Only participants with parental ratings of gross and fine motor function, perception, attention, and impulse control were included in the next phase of classification (590 girls and 566 boys). Furthermore, the attention performance was controlled because it influences the performance of impulse control. Therefore, participants with attention scores exceeding 2.5 were excluded based on the Z-score and 584 girls and 559 boys were kept at this stage. The present study further randomly selected participants (by way of drawing lots) to enable the same number of participants for each grade into each Z-score band and gender subgroup. In total, 710 participants (355 girls and 355 boys) were included after this processing.

To avoid the confounding effect caused by the overlapping of age among grades, a clear cutoff point of age was made between each adjacent grade. The youngest and oldest ages of participants in each grade were consistently 2 months younger and older than the previous and next grades, respectively. Thus, the data from children whose age overlapped with that of children in previous and next grades were eliminated. Then the aforementioned procedure for gender and Z-score band distribution among grade groups was repeated. Finally, 550 participants (285 girls and 265 boys) were included in the present study. Each grade included 110 children with 53 boys and 57 girls. The children’s age ranged from 82 to 142 months: grade 1 (G1; 88.00 ± 3.45 months), grade 2 (G2; 99.97 ± 3.00 months), grade 3 (G3; 111.11 ± 3.19 months), grade 4 (G4; 122.98 ± 3.30 months), and grade 5 (G5; 135.06 ± 3.70 months).

### 2.2. Measure of Impulsive Behavior: Dependent Variable

A total of nine questionnaire items, representing the nine core symptoms of impulsivity, were adopted from the Chinese version of the fourth edition of the Diagnostic and Statistical Manual of Mental Disorders (DSM-IV) [34,35]. The items are outlined as follows: (1) fidget with the hands or feet or squirm in a seat, (2) get up from the seat when expected to remain seated, (3) run or jump when and where it is inappropriate, (4) have trouble enjoying leisure activities or playing quietly, (5) often on the go or acts as if driven by a motor, (6) talk excessively, (7) blurt out answers before questions have been finished, (8) have trouble waiting for one’s turn when playing or other activities, and (9) interrupt or intrude others. On the questionnaire, the severity of impulsivity symptoms was rated on a 5-point scale (0 = always; 1 = often; 2 = sometimes; 3 = rarely; and 4 = never). The average score of all items was used as the performance index. This calculation was also used in following controlled variables: attention, fine motor, gross motor, and perception. 

The questionnaire on impulsivity was applied to typically and atypically developed children in grades 1–9. It yielded a test–retest coefficient of 0.51 over a period of 6 months (N = 97) and internal consistency of 0.91 (N = 1822) in typically developed children in grades 1–3. Moreover, its test–retest coefficient over a period of 6 months and its internal consistency were 0.74 (N = 96) and 0.90 (N = 1819), respectively, in typically developed children in grades 4–6. This questionnaire was also used in atypically developed children in grades 1–9, revealing an interprofessional reliability of 0.65 (N = 61) and a father–mother reliability of 0.75 (N = 92) [34]. 

The validity of group differences was established through significant one-way analysis of variance (ANOVA) findings, followed by post hoc analysis in children with typical development (N = 164), autism (N = 52), and attention deficit hyperactivity disorder (ADHD; N = 67). Children with typical development showed less impulsivity than did those with autism; children with autism demonstrated less impulsivity than did those with ADHD [34]. 

### 2.3. Measure of Inattention: Major Control Variable

Similar to the data on impulsive behavior items, nine items, representing the nine core symptoms of inattention, were adopted from the parent questionnaire in the Chinese version of inattention in the DSM-IV [34,35]. The items are outlined as follows: (1) make careless mistakes in schoolwork or other activities; (2) have trouble focusing on academic tasks or play activities; (3) do not seem to listen when spoken to directly; (4) fail to understand instructions; (5) fail to finish schoolwork or do not follow instructions; (6) have trouble organizing academic, play, or other activities; (7) avoid, dislike, or do not want to do things that require considerable mental effort for a long period; (8) lose things required for academic tasks and play activities; and (9) get distracted in or easily forget daily activities. Similar to impulsivity, inattention was measured on a 5-point scale (0 = always; 1 = often; 2 = sometimes; 3 = rarely; and 4 = never).

The questionnaire on inattention was used in typically and atypically developed children in grades 1–9. It yielded a test–retest coefficient of 0.60 over a period of 6 months (N = 97) and internal consistency of 0.91 (N = 1822) in typical children in grades 1–3. Its test–retest coefficient over a period of 6 months and its internal consistency were 0.64 (N = 96) and 0.90 (N = 1819), respectively, in typical children in grades 4–6. This questionnaire was also used in atypically developed children in grades 1–9, yielding an interprofessional reliability of 0.76 (N = 59) and a father–mother reliability of 0.73 (N = 91) [34].

The validity of group differences was established through significant one-way ANOVA findings, followed by post hoc analysis in children with typical development (N = 164), autism (N = 52), and ADHD (N = 67). Children with typical development showed less inattention than did those with ADHD, and children with ADHD demonstrated less impulsivity than did those with autism [34].

### 2.4. Performance of Fine and Gross Motor Function and Perception: Other Control Variables

The questionnaires of fine and gross motor function and perception applied 4-point scales to determine the severity of the corresponding items (0 = not at all; 1 = slight; 2 = medium; and 3 = serious). To exclude uncertain data and acquire certain data, more points were added (4 = observed but not sure; 5 = never observed; and 6 = not yet learned). The data were not analyzed if the respondent selected points 4–6 [35].

#### 2.4.1. Measure of Fine Motor Skills

The Contextual Fine Motor Questionnaire (CFMQ) was used to survey the participants’ fine motor performance. The questions in the CFMQ were concerned with handwriting (six items), art and craft activities (six items), the use of dining utensils (four items), tying knots (two items), managing buttons and zippers (four items), opening containers and packages (three items), strength (two items), and using hands often (one item) [33,35]. The detailed items are shown in Appendix A. 

#### 2.4.2. Measure of Gross Motor Skills

The Contextual Gross Motor Questionnaire (CGMQ) was used to survey the participants’ gross motor performance. The questions in the CGMQ were divided into 9 categories and 27 detailed items (Appendix B) [36]. This scale has not yet been reported formally. The categories are outlined as follows: (1) sitting posture (four items), (2) walking and running (five items), (3) climbing stairs (two items), (4) throwing and catching a ball (four items), (5) jumping (four items), (6) climbing (two items), (7) exercising and dancing (two items), (8) activities of daily living (two items), and (9) cycling (two items).

#### 2.4.3. Perception

The Contextual Visual Perception Questionnaire (CVPQ) was used to survey the participants’ visual perception performance [35,36]. The questions in the CVPQ assessed children’s global visual perception, involving playing with bricks and arranging tables and chairs (four items), solving puzzle activities (five items), copying figures (three items), identifying colors and shapes (four items), distinguishing between left and right (four items), wearing clothes (two items), and ability to find ways and determine directions (four items). The detailed items are shown in Appendix C.

## 3. Results

### 3.1. Reliability Check of the Present Data

Split-half reliability coefficients were computed for the main dependent variables (fine and gross motor function, perception, attention, and impulsive behavior) by correlating data for the entire sample with those for each group separately. The variable coefficients of the entire sample were consistently positive and high. The correlation for the items of fine and gross motor function and perception was strengthened (*r* = 0.846), as was that for attention and impulsive behavior (*r* = 0.801), suggesting the robustness of the survey procedure across all grades. 

### 3.2. Check of Variables among Different Grade Groups

Moreover, ANOVA was performed across behavioral performance to examine the effects of grade on the variables (fine and gross motor function, perception, attention, and impulsive behavior). Only the impulsive behavior items showed significant variations for each grade (*p* < 0.001) and gender (*p* < 0.001). For the purpose of controlling the effect of gender on impulse control, the Z-scores of female and male were computed respectively and used separately. Consequently, the impulsive behavior score as well as other variables between genders was not significantly different. Therefore, we decided to use Z-scores for all variables according to gender (Table 1).

### 3.3. Developmental Differences in Impulsive Behavior

To conduct the homogenous data analysis, the matched groups were classified by the Z-scores distribution (based on the means and standard deviations regarding the attention item). The performance analysis did not indicate significant differences in attention, fine motor function, gross motor function, and perception showing that these four variables were controlled statistically (Table 1). However, significant differences were observed in impulsive behavior (*p* < 0.001). Next, statistical analyses were conducted to compare the differences in impulsive behavior among grades. The post hoc comparison results indicated significant differences between G1 and G5 (*p* < 0.001) as well as between G2 and G5 (*p* < 0.001) (Table 2).

## 4. Discussion

The present survey study explored the characteristics of developmental differences in impulse control behavior in school children aged 82–142 months, after controlling for potential confounding variables associated with impulse control in the attention and age of participants. Furthermore, only items assessing impulse control in the questionnaire were associated with significant developmental time courses both between G5 (10.83–11.83 years) and G1 (6.83–7.75 years; *p* < 0.001) as well as G2 (7.92–8.67 years; *p* < 0.001). The main findings of the present study indicate that the impulse control abilities in older children are more stable with developmental improvements than are those in younger children. The impulse control center is considered to be located in the frontal lobe of the brain [15,37]. At 10 years, the impulse control matures with the improved function of the frontal lobe [23,38]. Thus, the frontal lobe does not completely mature until after the age of 10 years [10,22,39]. This finding regarding developmental improvement has been reported by empirical studies on perceptual–motor performance in school-age children.

Compared with lower (G1 and G2) and higher (G5) grades, no significant developmental differences in impulse control were observed in middle grades (G3 and G4) in the present study. The middle grades, which succeed from G2 and proceeded to G5, seem to play a key role as the transitional stage. Previously studies have reported two striking developmental advancements in impulse control during elementary school. The first developmental phase occurs at age 7–8 years, corresponding to G1 (6.83–7.75 years), and the other period occurs at age 9–12 years, corresponding to G3–G5 (8.83–11.83 years) in this study [23,24]. Although studies have analyzed children in G3–G5, the present results reveal that the abilities of impulse control distinctively vary among these grades. This finding is supported by the absence of significant differences in statistical comparisons between children in the lower and middle grades. The present findings indicate that the middle grades might serve as the transitional stage of the development of impulse control. This stage might play a critical role not only in increasing age but also in impulse control behavior during elementary school years. Considering the maturation of impulse control by the age of 10 years, corresponding to G4 in the present study [23,38], the academic grade stratification provides an opportunity to understand the clarified transitional trend of impulse control in higher grades (G5) as well as its actual developmental shifting in lower grades (G1 and G2). Typically, the function of impulse control has been considered to improve with age along with the development of the prefrontal cortex [15,38].

The grade-dependent changes in this survey study provide stratified information on developmental traits of impulse control in school-age children. Our finding indicates that the competence of impulse control improved with age in the examined children. Therefore, as indicated by the present study, over the entire age range of our sample, older children (those in high grades) showed more effective impulse control behavior than did younger children (those in low grades). The observation of developmental improvement in impulse control is in agreement with those reported in a previous study that involved a response inhibition task [10] and is consistent with substantial evidence demonstrating that children typically become less impulsive with age [9,10]. Moreover, according to the post hoc analysis, the developmental trait of impulse control developed stagewise in the present study which examined the relationships of developmental traits throughout the elementary period to understand the characteristics of impulsive behavior in school-age children. The post hoc analysis revealed significant differences between various grades, thus indicating significant differences in the relationships between grade and impulsive behavior. According to the observed developmental trait, the grades were categorized into three groups: G1 and G2 to group 1, G3 and G4 to group 2, and G5 to group 3. This stratified relationship between grades and impulsive behavior was a significant predictor of inhibitory abilities and the later development of impulse control function in children. Compared with previous studies, the striking developmental advancements in impulse control first occurred at age 6–7.5 years [23], which corresponds to group 1 (age, 6.92–7.84 years) in the present study. The next developmental improvement in impulsive errors and missed responses was found between 7–8-years old and 9–12-years old [10,23,24], which, in concordance with previous studies, are consistent with group 2 (age, 7.76–10.56 years) and group 3 (age, 10.93–11.57 years) in this study, respectively. In summary, impulsive behavior was completely developed by the age of 10 years, with little further improvement in group 3 [24].

## 5. Limitations and Future Studies

A possible limitation of this survey study is that we determined the developmental trait in association with performance components instead of performance areas. The bottom-up approach revealed significant relationships between the development of performance components, such as attention and gross and fine motor function, and impulse control behavior. The bottom-up approach is perhaps more efficient in controlling for confounding factors; however, this has the limitation that any resulting between the components can only provide the information as good as the original conceptualization of performance. Impulse control behavior has a critical influence on the development of functional and purposeful capacities including, but not limited to, behavioral, social, emotional, and academic regulations in school children. Through the use of the top-down approach, the relationships between impulse control and occupational performances (or aforementioned functions) can be comprehended through the development of the adaptive interaction of tasks; this thus warrants further study.

Moreover, impulse control has been connected with executive function [40,41]. In the clinical practice, the concepts of executive function are widely applied and currently merged with therapeutic programs [42,43]. It can be considered that a further survey, associated with laboratory testing of the interaction between impulse control and executive function, will provide the opportunity for clinical professionals to develop a more comprehensive executive functioning program.

The data of this study derived from an existed database established by survey. Therefore, the design and instruments of this study, as well as the way of excluding children who were suspected to be experiencing developmental delay, might be limited. In the future, a prospective research design based on objective and accurate behavioral assessments are suggested.

## 6. Conclusions

The result of the analysis of developmental traits in this study represents impulse control functioning in typical school children and indicates that a striking development of impulse control occurs from grade 4 to 5, which is similar to those reported previously [23]. It strongly supports the effect of age on the function of impulse control without the confounding influences of motor, attention and perception performance. Knowledge of such developmental stability and change in normal function is important for analyzing and understanding behavioral performance levels and impulse control function associated with various developmental disorders, particularly when considering whether clinical disorders and problems represent difficulties in teaching and learning programming, or when investigating favorable parent–child relationships.

## Figures and Tables

**Table 1:** ANOVA Performed Across Behavioral Performance to Examine the Effects of Grade on the Dependent Variables.

Measure	Group						
	G1	G2	G3	G4	G5		
	M	M	M	M	M	*F*	*P*
	(SD)	(SD)	(SD)	(SD)	(SD)		
Attention	0.07	0.40	0.07	0.04	0.09	0.066	0.992
	(0.88)	(0.90)	(0.08)	(0.87)	(0.90)		
Fine Motor	0.04	0.04	−0.04	−0.07	−0.23	1.876	0.113
	(0.88)	(0.96)	(0.78)	(0.82)	(0.74)		
Gross Motor	−0.16	−0.03	0.04	−0.05	−0.04	0.827	0.508
	(0.60)	(0.84)	(0.94)	(0.85)	(0.89)		
Perception	−0.16	−0.06	−0.03	−0.04	−0.08	0.470	0.758
	(0.60)	(0.90)	(0.77)	(0.85)	(0.88)		
Inhibition	0.22	0.22	−0.01	−0.04	−0.32	6.925	0.000
	(1.04)	(0.93)	(0.89)	(0.83)	(0.75)		

**Table 2:** Post hoc comparisons using the Tukey Method for Inhibition Score at the 0.05 level of significance.

Grade (i)	Grade (j)	Difference in Aver (i–j)	Standard Error	*p*
1	2	0.007	0.125	1.000
	3	0.232	0.124	0.335
	4	0.269	0.124	0.193
	5	0.552 (***)	0.125	0.000
2	1	−0.007	0.125	1.000
	3	0.225	0.124	0.371
	4	0.262	0.124	0.219
	5	0.545 (***)	0.125	0.000
3	1	−0.232	0.124	0.335
	2	−0.225	0.124	0.371
	4	0.037	0.124	0.998
	5	0.320	0.124	0.076
4	1	−0.269	0.124	0.193
	2	−0.262	0.124	0.219
	3	−0.037	0.124	0.998
	5	0.283	0.124	0.154
5	1	−0.552 (***)	0.125	0.000
	2	−0.545 (***)	0.125	0.000
	3	−0.320	0.124	0.076
	4	−0.283	0.124	0.154

Note: *** *p* < 0.001.

## Data Availability

There is no data statement online for this article.

## Appendix A

**Table A1:** The questionnaire of fine motor performance.

About Children’s Performance on Hand Movements (Fine Movements/Hand Functions)		Not at All	Slight	Medium	Serious	Observed but Not Sure	Never Observed	Not Yet Learned
1. Hand-writing	a. Does he write too slowly because of poor hand movements?							
	b. Is his handwriting too scratchy because of poor hand movements?							
	c. Does he fail to produce straight lines when writing (for example: as if he is writing with a shaking hand, or is unable to control the hand to make the line straight)?							
	d. Does he crumple up or pierce the paper because of pressing the pen too hard?							
	e. Is his handwriting unclear because he writes too softly?							
	f. Is the way that he holds the pen awkward?							
2. Arts and Crafts	a. Does he use the scissors clumsily (for examples: getting paper jammed, or unable to make smooth movements)?							
	b. Is the finished article cut untidily (for example: the article isn’t cut according to the lines)?							
	c. Does he often drop the materials or tools when working on handicrafts?							
	d. Does he often mess things up when using the glue?							
	e. Does he work on handicrafts very slowly?							
	f. Is the finished handicraft of poor quality (twisted around/wrinkled)?							
3. The Use of Dining Utensils	a. Is he eating too slowly because of poorly coordinated hand movements?							
	b. Does he often drop the food when using chopsticks?							
	c. Does he often drop the food when using a spoon?							
	d. Does he often drop spoons, chopsticks, bowls or dishes?							
4. Tying Knots	a. Does he have any difficulty in tying up shoelaces because of poor hand movements?							
	b. Does he have any difficulty in tying knots because of poor hand movements?							
5. Buckling and Zipping Up	a. Does he have any difficulty in buckling up because of poor hand movements?							
	b. Does he have any difficulty in unbuckling because of poor hand movements?							
	c. Does he have any difficulty in inserting the zipper opening into the slider because of poor hand movements?							
	d. After inserting the zipper opening to the slider, does he have any difficulty in zipping up (one hand holding the end of zipper and the other pulling the slider) because of poor hand movements?							
6. Opening Containers	a. Does he have any difficulty in opening packs (for examples: snack packs, candy wraps, biscuit packing, milk boxes, parcels, etc.)?							
	b. Does he have any difficulty in opening easy-open cans?							
	c. Does he have any difficulty in opening bottles?							
7. Strength	a. Are his arms too weak (for example, when playing arm wrestling or the horizontal bar)?							
	b. Are his hands or fingers too weak (for example, when squeezing a towel out)?							
8. Handed-ness	a. During an activity, does the child often switch hands and obstruct the smoothness of actions?							

Note. Twenty seven items of this scale were used to conduct factor analysis after removing the last item (handedness) [33]. In this present study, we used those original 28 items to represent fine motor performance [35].

## Appendix B

**Table A2:** The questionnaire on gross motor performance.

About Children’s Gross Movements		Not at All	Slight	Medium	Serious	Observed but Not Sure	Never Observed	Not Yet Learned
1. Sitting Posture	a. Is his sitting posture awkward?							
	b. Is his standing posture awkward?							
	c. Is his body feeble? (looks weak or often leans against something)							
	d. Does he often lie on the desk?							
2. Walking and Running	a. Does he often trip over when he walks?							
	b. Does he often bump into people or things when he walks?							
	c. Does he often trip over when he runs?							
	d. Does he often bump into people or things when he runs?							
	e. Does he often lag behind when running with his peers?							
3. Climbing Stairs	a. Does he often trip over, bump into people or slow down when climbing stairs?							
	b. Does he often trip over, bump into people or slow down when descending stairs?							
4. Throw-ing and Catching Balls	a. 1. Is his posture of throwing balls awkward or poorly coordinated?							
	b. 2. Is he unable to aim the target when throwing balls?							
	c. 3. Is his posture when catching balls awkward or poorly-coordinated?							
	d. 4. Does he often fail to catch the ball?							
5. Jumping	a. 1. Is his jumping posture awkward or poorly-coordinated?							
	b. Is he unable to jump long distances?							
	c. Is he unable to jump high?							
	d. Does he take a long time to learn, or fail to learn, rope skipping?							
6. Climbing	a. Does he lag behind his peers when playing with climbing facilities in playground?							
	b. Is he unskillful when playing with climbing facilities in playground?							
7. Exercise/ Dancing	a. Is he unable to follow the instructor or the rhythm when doing exercise/dancing?							
	b. Does he imitate the steps of exercise or dance incorrectly?							
8. Daily Living	a. Does he often spill the water when carrying a washbowl or bucket?							
	b. Is he unable to coordinate his movements when sweeping the floor?							
9. Cycling	a. Does he fail to learn or take a longer time to learn cycling as compared to his peers?							
	b. Does he always dodder, bump into something or fall down when he rides a bicycle?							

## Appendix C

**Table A3:** The questionnaire on perception performance.

About Children’s Performance on Perception		Not at All	Slight	Medium	Serious	Observed but Not Sure	Never Observed	Not Yet Learned
1. Playing with Bricks and Arranging Tables/Chairs	a. Does he hate playing with bricks?							
	b. Does he join bricks very slowly?							
	c. Is he unable to join bricks accurately according to the illustration. (Is the finished product different from the illustration)?							
	d. Does he arrange tables, chairs, and teaching tools inaccurately?							
2. Doing Puzzles	a. Does he hate playing with puzzles?							
	b. Does he play with puzzles very slowly?							
	c. Does he take a long time to restore or notice the proper order of shuffled jigsaws?							
	d. Does he always piece together the wrong jigsaw pieces?							
	e. Is he unable to restore shuffled jigsaws?							
3. Copying Figures	a. Does he hate copying figures?							
	b. Does he copy figures very slowly?							
	c. Does he fail to draw figures correctly according to the illustration. (Is the finished drawing different from the illustration)?							
4. Identifying Colors and Shapes	a. Does he take a longer time to learn different shapes (for examples: triangle, square, etc.) compared to other children?							
	b. Does he lag behind his peers in recognizing figures?							
	c. Does he take a longer time to learn different colors compared to his peers?							
	d. Does he lag behind his peers in recognizing colors?							
5. Distinguish-ing between Left and Right	a. Does he often turn in the wrong direction when making a right or left turn?							
	b. When asked to raise up the right or left hand, does the child raise the wrong hand?							
	c. Does he write a word in a reverse order? If so, please give an example:							
	d. Overall, does the child have any difficulty in distinguishing between left and right?							
6. Wearing Clothes	a. When he puts on a pair of shoes, does he mix up the left and right shoes?							
	b. Does he put on clothes back to front, inside out, or mixing up the left and right?							
7. Sense of Direction	a. Does he have a poor sense of direction?							
	b. Is he often confused with directions on the road (for example: when going out)?							
	c. Does he often get lost?							
	d. Is he unable to follow the same route on return?							

## References

[1] Eysenck S.B., Eysenck H.J. (1977). The place of impulsiveness in a dimensional system of personality description. Br. J. Soc. Clin. Psychol..

[2] Blair C., Raver C.C. (2015). School readiness and self-regulation: A developmental psychobiological approach. Annu. Rev. Psychol..

[3] Ketch K.M., Brodeur D.A., McGee R. (2009). The effects of focused attention on inhibition and state regulation in children with and without attention deficit hyperactivity disorder. J. Appl. Dev. Psychol..

[4] Moffitt T.E., Arseneault L., Belsky D., Dickson N., Hancox R.J., Harrington H., Houts R., Poulton R., Roberts B.W., Ross S. (2011). A gradient of childhood self-control predicts health, wealth, and public safety. Proc. Natl. Acad. Sci. USA.

[5] Potenza M.N., Koran L.M., Pallanti S. (2009). The relationship between impulse-control disorders and obsessive-compulsive disorder: A current understanding and future research directions. Psychiatry Res..

[6] van Lier P.A., Muthén B.O., van der Sar R.M., Crijnen A.A. (2004). Preventing disruptive behavior in elementary schoolchildren: Impact of a universal classroom-based intervention. J. Consult Clin. Psychol..

[7] Blair C., Diamond A. (2008). Biological processes in prevention and intervention: The promotion of self-regulation as a means of preventing school failure. Dev. Psychopathol..

[8] Montroy J.J., Bowles R.P., Skibbe L.E., McClelland M.M., Morrison F.J. (2016). The development of self-regulation across early childhood. Dev. Psychol..

[9] Paulsen K., Johnson M. (1980). Impulsivity: A multidimensional concept with developmental aspects. J. Abnorm. Child. Psychol..

[10] Williams B.R., Ponesse J.S., Schachar R.J., Logan G.D., Tannock R. (1999). Development of Inhibitory Control Across the Life Span. Dev. Psychol..

[11] Marsh R., Zhu H., Schultz R.T., Quackenbush G., Royal J., Skudlarski P., Peterson B.S. (2006). A developmental fMRI study of self-regulatory control. Hum. Brain Mapp..

[12] Posner M.I., Rothbart M.K., Sheese B.E., Tang Y. (2007). The anterior cingulate gyrus and the mechanism of self-regulation. Cogn. Affect Behav. Neurosci..

[13] Adleman N.E., Menon V., Blasey C.M., White C.D., Warsofsky I.S., Glover G.H., Reiss A.L. (2002). A developmental fMRI study of the Stroop color-word task. Neuroimage.

[14] Pliszka S.R., Liotti M., Woldorff M.G. (2000). Inhibitory control in children with attention-deficit/hyperactivity disorder: Event-related potentials identify the processing component and timing of an impaired right-frontal response-inhibition mechanism. Biol. Psychiatry.

[15] Boes A.D., Bechara A., Tranel D., Anderson S.W., Richman L., Nopoulos P. (2009). Right ventromedial prefrontal cortex: A neuroanatomical correlate of impulse control in boys. Soc. Cogn. Affect Neurosci..

[16] Casey B.J., Galvan A., Hare T.A. (2005). Changes in cerebral functional organization during cognitive development. Curr. Opin. Neurobiol..

[17] Bunge S.A., Dudukovic N.M., Thomason M.E., Vaidya C.J., Gabrieli J.D. (2002). Immature frontal lobe contributions to cognitive control in children: Evidence from fMRI. Neuron.

[18] Blair C. (2003). Behavioral inhibition and behavioral activation in young children: Relations with self-regulation and adaptation to preschool in children attending head start. Dev. Psychobiol..

[19] Bronson M.B. (2000). Recognizing and supporting the development of self-regulation in young children. Young Child..

[20] Kochanska G., Coy K.C., Murray K.T. (2001). The development of self-regulation in the first four years of life. Child. Dev..

[21] Loeber R., Green S.M., Lahey B.B., Frick P.J., McBurnett K. (2000). Findings on disruptive behavior disorders from the first decade of the developmental trends study. Clin. Child. Fam. Psychol. Rev..

[22] Luna B., Marek S., Larsen B., Tervo-Clemmens B., Chahal R. (2015). An integrative model of the maturation of cognitive control. Annu. Rev. Neurosci..

[23] Brocki K.C., Bohlin G. (2004). Executive functions in children aged 6 to 13: A dimensional and developmental study. Dev. Neuropsychol..

[24] Levin H.S., Culhane K.A., Hartmann J., Evankovich K., Mattson A.J., Harward H., Ringholz G., Ewing-Cobbs L., Fletcher J.M. (1991). Developmental changes in performance on tests of purported frontal lobe functioning. Dev. Neuropsychol..

[25] Henderson H.A., Pine D.S., Fox N.A. (2015). Behavioral inhibition and developmental risk: A dual-processing perspective. Neuropsychopharmacology.

[26] Winstanley C.A., Eagle D.M., Robbins T.W. (2006). Behavioral models of impulsivity in relation to ADHD: Translation between clinical and preclinical studies. Clin. Psychol. Rev..

[27] Börger N., van der Meere J. (2000). Motor control and state regulation in children with ADHD: A cardiac response study. Biol. Psychol..

[28] Pitcher T.M., Piek J.P., Hay D.A. (2003). Fine and gross motor ability in males with ADHD. Dev. Med. Child. Neurol..

[29] Zimmerman B.J., Martinez-Pons M. (1990). Student differences in self-regulated learning: Relating grade, sex, and giftedness to self-efficacy and strategy use. J. Educ. Psychol..

[30] Gillberg C. (2003). Deficits in attention, motor control, and perception: A brief review. Arch. Dis. Child..

[31] Eisenberg N., Guthrie I.K., Fabes R.A., Shepard S., Losoya S., Murphy B.C., Jones S., Poulin R., Reiser M. (2000). Prediction of elementary school children’s externalizing problem behaviors from attentional and behavioral regulation and negative emotionality. Child. Dev..

[32] Leblanc N., Boivin M., Dionne G., Brendgen M., Vitaro F., Tremblay R.E., Pérusse D. (2008). The development of hyperactive-impulsive behaviors during the preschool years: The predictive validity of parental assessments. J. Abnorm. Child. Psychol..

[33] Lin C.K., Meng L.F., Yu Y.W., Chen C.K., Li K.H. (2014). Factor analysis of the Contextual Fine Motor Questionnaire in children. Res. Dev. Disabil..

[34] Hung L.Y., Chiu Y.N., Chang Y.W., Meng Y.J., Tsai M.F. (2001). The 3rd year end-of-term report of work on the diagnosis, placement and consultation of ADHD students: A research report commissioned by Special Education Unit in the Ministry of Education, Taiwan.

[35] Tsai L.H., Meng L.F., Hung L.Y., Chen H.Y., Lu C.P. (2011). Coincidence of homophone spelling errors and attention problems in schoolchildren: A survey study. Res. Dev. Disabil..

[36] Meng L.F. (2004). Do children with developmental coordination disorder comorbid for sensory and perceptual difficulties. National Science Council Report of Taiwan.

[37] Berlin H.A., Rolls E.T., Kischka U. (2004). Impulsivity, time perception, emotion and reinforcement sensitivity in patients with orbitofrontal cortex lesions. Brain.

[38] Welsh M.C., Pennington B.F., Groisser D.B. (1991). A normative-developmental study of executive function: A window on prefrontal function in children. Dev. Neuropsychol..

[39] Bezdjian S., Baker L.A., Lozano D.I., Raine A. (2009). Assessing inattention and impulsivity in children during the Go/NoGo task. Br. J. Dev. Psychol..

[40] Böhm B., Smedler A.-C., Forssberg H. (2004). Impulse control, working memory and other executive functions in preterm children when starting school. Acta Paediatr. Int. J. Paediatr..

[41] Leshem R., De Fano A., Ben-Soussan T.D. (2020). The implications of motor and cognitive inhibition for hot and cool executive functions: The case of quadrato motor training. Front. Psychol..

[42] Hecht M.F., Garber C.E. (2021). Effectiveness of the POWER program in Improving physical activity and executive function in fifth grade students. J. School Health.

[43] Pouretemad H., Shiri E. (2021). Improvement of executive function in children with autism through home playtime program. Koomesh.

